# Robustness analysis of the detailed kinetic model of an ErbB signaling network by using dynamic sensitivity

**DOI:** 10.1371/journal.pone.0178250

**Published:** 2017-05-24

**Authors:** Hiroyuki Masunaga, Yurie Sugimoto, Shigeyuki Magi, Ryunosuke Itasaki, Mariko Okada-Hatakeyama, Hiroyuki Kurata

**Affiliations:** 1 Department of Bioscience and Bioinformatics, Kyushu Institute of Technology, Iizuka, Fukuoka, Japan; 2 Laboratory for Integrated Cellular Systems, RIKEN Center for Integrative Medical Sciences (IMS), Tsurumi-ku, Yokohama, Kanagawa, Japan; 3 Laboratory of Cell Systems, Institute for Protein Research, Osaka University, Suita-shi, Osaka, Japan; 4 Biomedical Informatics R&D Center, Kyushu Institute of Technology, Iizuka, Fukuoka, Japan; Emory University Winship Cancer Institute, UNITED STATES

## Abstract

The ErbB receptor signaling pathway plays an important role in the regulation of cellular proliferation, survival and differentiation, and dysregulation of the pathway is linked to various types of human cancer. Mathematical models have been developed as a practical complementary approach to deciphering the complexity of ErbB receptor signaling and elucidating how the pathways discriminate between ligands to induce different cell fates. In this study, we developed a simulator to accurately calculate the dynamic sensitivity of extracellular-signal-regulated kinase (ERK) activity (ERK*) and Akt activity (Akt*), downstream of the ErbB receptors stimulated with epidermal growth factor (EGF) and heregulin (HRG). To demonstrate the feasibility of this simulator, we estimated how the reactions critically responsible for ERK* and Akt* change with time and in response to different doses of EGF and HRG, and predicted that only a small number of reactions determine ERK* and Akt*. ERK* increased steeply with increasing HRG dose until saturation, while showing a gently rising response to EGF. Akt* had a gradual wide-range response to HRG and a blunt response to EGF. Akt* was sensitive to perturbations of intracellular kinetics, while ERK* was more robust due to multiple, negative feedback loops. Overall, the simulator predicted reactions that were critically responsible for ERK* and Akt* in response to the dose of EGF and HRG, illustrated the response characteristics of ERK* and Akt*, and estimated mechanisms for generating robustness in the ErbB signaling network.

## Introduction

The ErbB receptor signaling network is highly interconnected and regulates diverse responses in a variety of cells and tissues. Dysregulation of the network is responsible for the development and progression of several types of human cancer [1]. In MCF-7 human breast cancer cells, stimulation with epidermal growth factor (EGF), a ligand for the epidermal growth factor receptor (EGFR), or heregulin (HRG), a ligand for ErbB3/ErbB4 receptors, induces transient or sustained activity of intracellular kinases, depending on the ligand concentrations [2]. In particular, sustained and transient extracellular-signal-regulated kinase (ERK) activity (ERK*) or Akt activity (Akt*) is known to induce differentiation and proliferation of MCF-7 cells, respectively [3], indicating that duration and sustainability of kinase activity is important to determine cell fates. Thus, a quantitative understanding of ErbB receptor signaling, and the regulatory mechanisms underlying the dynamics of the network, is important to establish effective strategies for treating cancers driven by network dysregulation.

The multiple interconnecting pathways and feedback loops involved in ErbB signaling make it difficult to predict the dynamic responses of the network. In this regard, mathematical modelling is an attractive approach to predicting dynamic behaviors under different conditions, and understanding how a system responds to input signals and different kinds of perturbations. Accordingly, mathematical modeling approaches have been applied to analyze EGFR/ErbB signaling dynamics and identify underlying molecular mechanisms (Kholodenko et al.(1999)[4], Schoeberl et al.(2002)[5], Hatakeyama et al.(2003)[6], Hendriks et al.(2003)[7], Resat et al.(2003)[8], Blinov et al.(2006)[9], Shankaran et al.(2006)[10], Birtwistle et al.[11], and Nakakuki et al.[3]). Although network architecture, such as feedback and feedforward loops, reflects some of the mechanisms that generate robustness and output properties, it does not address quantitative interpretations. Kinetic models are required to estimate the contribution of each pathway to the properties and phenotypes of the network.

Sensitivity analysis can identify critical reactions and estimate robustness of a biochemical network. Single parameter sensitivity is used to perform a local sensitivity analysis in static or dynamic ways. Static sensitivity analysis provides steady-state insight, while dynamic sensitivity (DS) analyzes time-variation modalities such as transient and oscillatory systems [12]. DS analysis can be roughly divided into the direct differential methods (DDMs) [13] and the indirect differential methods (IDMs) [14,15]. The DDMs solve the ordinary differential equations and their associated DS equations simultaneously, where the DSs are described in symbolic form. The IDMs infinitesimally perturb the value of one specific parameter, while keeping the other parameters constant; thus the simulation results contain approximation errors. Global sensitivity analysis quantifies the sensitivities of the model outputs with respect to variations of multiple parameters. To date, sampling-based and variance-based methods have been proposed based on random sampling and Monte-Carlo integrations [16]. Since there is generally a tradeoff between calculation speed and accuracy, the choice of method depends on the requirements of model size and nonlinearity. From the many options, multi-parameter sensitivity (MPS) [17], the sum of the squared magnitudes of single-parameter sensitivities, is practical in terms of theoretical background, applicability to biology, and computational cost. MPS represents how a system’s output varies when small, random, and simultaneous fluctuations are provided to many kinetic parameters.

In this study, we developed a simulator to calculate the dynamic sensitivity of ERK* and Akt* in an ErbB signaling network model with 237 kinetic parameters using MCF7 breast cancer cells. To demonstrate the feasibility of this simulator, we predicted reactions that were critically responsible for ERK* and Akt* in response to the dose of EGF and HRG, illustrated the response characteristics of ERK* and Akt*, and estimated mechanisms for generating robustness in the ErbB signaling network.

## Materials and methods

### ErbB signaling network model

**Fig 1:**
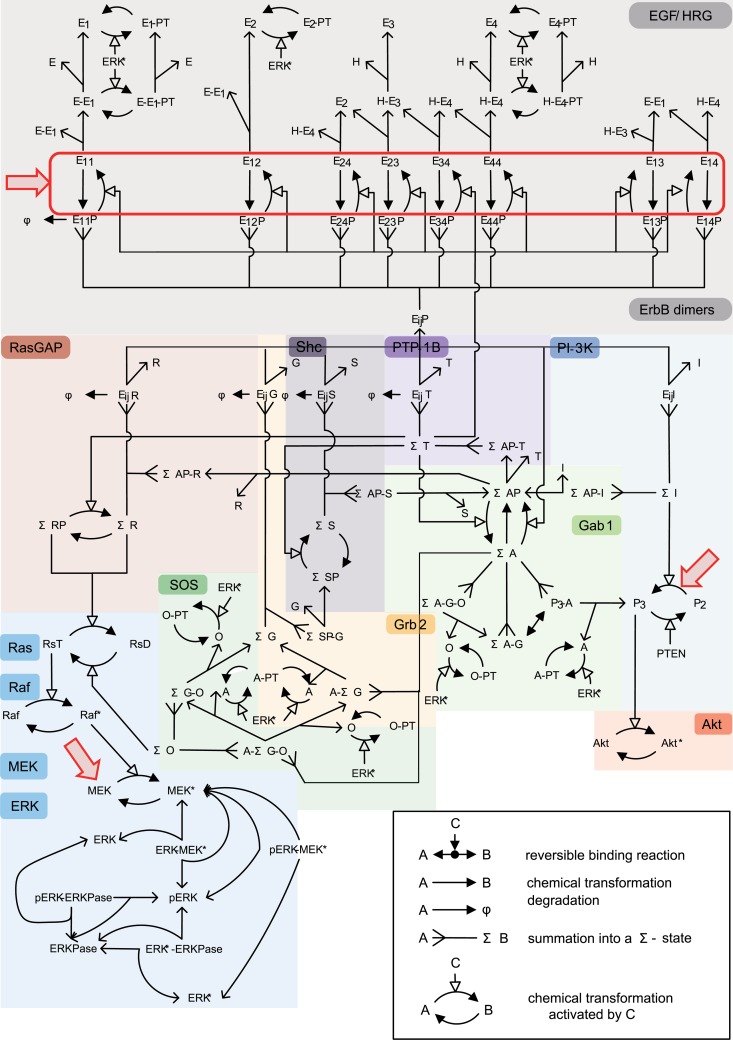
The MCF7 ErbB signaling network map. Σ-states are summations over specific membrane-localized species with identical downstream signaling activity and membrane-anchorage. ΣA:P3-A, ΣA-G, A-ΣG-O, ΣA-G-O, A-ΣG, ΣI:E_ij_I, ΣAP-I, ΣG:E_ij_G, ΣSP-G, ΣS:E_ij_S, ΣAP-S, ΣR:E_ij_, ΣAP-R, ΣT:E_ij_T, ΣAP-T, ΣO:ΣG-O, A-ΣG-O, ΣA-G-O, E:EGF, H:HRG, E_i_:ErbB, G:Grb2, S:Shc, I:PI3K, T:PTP1-B, O:SOS, A:Gab1, R:RasGAP, RsD:Ras-GDP, RsT:Ras-GTP, P2:PIP2, P3:PIP3, E_ij_:ErbB homo- or heterodimer bound, E_ij_X: E_ij_ bound to protein. Single-sided solid-head arrows with solid lines depict chemical transformation, while those with dotted lines depict a potentially multistep chemical reaction process. Single-sided double solid-head arrows depict summation into a Σ-state. P denotes tyrosine phosphorylation, PT denotes threonine/serine phosphorylation, and *denotes activation. Red arrows show the targets of ERK, Akt, and ErbB inhibitors.

The ErbB signaling cascade in MCF7 cells consists of the following components: extracellular ligands (EGF and HRG), four trans-membrane protein kinase receptors ErbB1 (EGFR), ErbB2 (HER2/NEU), ErbB3, and ErbB4, cytoplasmic adapter/scaffold proteins (Gab1, Grb2, and Shc), phosphatase (PTP-1B), enzymes/protein factors (PI-3K, SOS, and RasGAP), the Ras-Raf-MEK-ERK pathway, and small molecules (GTP) [1] (Fig 1). A ligand binding to a receptor leads to homo- or heterodimerization of the receptors. Single-molecule imaging analysis demonstrated that the binding constants of ErbB receptors to EGF and HRG are the same [18,19]. The receptor’s tyrosine kinase domain is activated by phosphorylation of tyrosine residues on cytoplasmic tails of the receptor by dimerization partners. The phosphorylated tyrosine residues of the receptors can bind to cytoplasmic adapter/scaffold proteins, and enzymes recruited to the plasma membrane. The interactions between these components finally activate multiple downstream proteins, including ERK and Akt, which play an important role in activation of transcription factors driving cell growth, survival, and differentiation.

We improved the kinetic model developed by Birtwistle et al. (2007) [11] to apply the DDM to the model (S1–S6 Tables). We removed the if-then rules from the original model to make the equations differentiable during the entire simulation time. The model contains the Ras/Raf /MAPK and PI-3K/Akt pathways, as well as multiple ERK-mediated feedback loops. It comprises 95 reactions with 126 molecular species and 237 kinetic parameters. The kinetic model is given by:

$$\frac{dx_{i}}{dt}=f_{i}(x,p)\;(i=1,2,\ldots ,Nx)$$ (1)

$$x_{i}=x_{0i}\;t=0$$ (2)

where *t* is the time, *i* is the index of molecular concentrations, **x** = (*x*_1_, *x*_2_, …, *x*_*Nk*_)^*T*^ is the vector of molecular (species) concentrations, *Nx* is the number of molecules, **p** = (*p*_1_, *p*_2_, …, *p*_*Np*_)^*T*^ is the vector of constant parameters, *Np* is the number of parameters, *f*_*i*_ is the mass balance function for *x*_*i*_, and *x*_0*i*_ is the initial value. The model investigates the dynamics until 1,800 s, assuming that the protein concentrations are kept constant. Since gene expression occurs after 1,800 s and protein concentrations change, it is difficult to build a model after 1,800 s due to the complexity of gene regulation. Thus, the model is applicable for the simulation time from 0 s to 1,800 s.

### Analysis flow

We performed the following investigations: (i) Simulation and analysis of the dynamic responses of ERK* and Akt* to systematic changes in EGF and HRG doses, (ii) Estimation of critical parameters by simulating single-parameter DSs, and (iii) Analysis of mechanisms by which ERK* and Akt* network show robustness to perturbations to intracellular kinetics and addition of inhibitors.

### Output properties

We used ERK* and Akt* at 1,800 s as the system's output. In general, the output properties or switching properties can be estimated by a response characteristic to input signals and by robustness to perturbations, characterized by sensitivity analysis.

### DDM for DS

Single parameter sensitivity is defined by:

$$s(x_{i},p_{j})=\frac{\partial x_{i}(t,p)}{\partial p_{j}}$$ (3)

The absolute DS of the differential equations is given by:

$$\frac{ds(x_{i},p_{j})}{dt}=\sum_{k=1}^{Nx}\frac{\partial f_{i}}{\partial x_{k}}s(x_{k},p_{j})+\frac{\partial f_{i}}{\partial p_{j}},$$ (4)

where *i* is the index of molecules and *j* is the index of parameters. The relative DS is given by:

$$S(x_{i},p_{j})=\frac{\partial x_{i}(t,p_{j})}{\partial p_{j}}\frac{p_{j}}{x_{i}(t,p_{j})}$$ (5)

In this study, we used the relative DS for all the simulations and analyses.

### MPS analysis

The MPS characterizing the total robustness is calculated as the sum of the squared magnitudes of single-parameter sensitivities [17].

$$MPS(x_{i},p)={\sum_{j=1}^{n}\left(\frac{\partial x_{i}(t,p_{j})}{\partial p_{j}}\frac{p_{j}}{x_{i}(t,p_{j})}\right)}^{2},$$ (6)

where *n* is the number of parameters and *MPS* (*x*_*i*_, **p**) is the target function or output for a system. MPS is a theoretical and intelligible measure that characterizes robustness much faster than the existing methods, based on the assumption that the relative change in the target function is linear in response to changes in each parameter. Actually, the MPS is a feasible measure for quantifying robustness in response to small perturbations in many biological, nonlinear models [17].

### Implementation

We developed the Matlab-based simulator to accurately calculate DSs by the DDM. The simulator with instructions is freely available at: http://www.cadlive.jp/cadlive_main/Softwares/DSsimulator/DynamicSensitivity.html. Since the simulator employs our own application of the partial differentiation converter [20, 21], it does not require any options other than the main body of Matlab. Numerical simulation and subsequent statistical analysis were performed by MATLAB^®^ R2013a version 8.1 (MathWorks 2013) on a personal computer using Windows 7 (CPU: Intel^®^ Core^™^ i7-2760QM 2.40 GHz, RAM: 8.00 GByte).

### Immunostaining and imaging cytometry experiments

MCF-7 cells were seeded at a density of 1 × 10^4^ cells/well in 96-well plates for fluorescent imaging. The next day, culture medium was replaced with serum free medium. After 16 h, cells were stimulated with EGF and HRG for the indicated period, fixed with 4% paraformaldehyde in PBS, and permeabilized with 0.1% Triton X-100 in PBS for 5 min. After washing with PBS, the cells were incubated in blocking buffer and then incubated with primary antibodies (anti-p-ERK (ERK*) antibody #4370, and anti-p-Akt (Akt*) antibody #2965 from Cell Signaling Technology) at 4°C. The next day the cells were fluorescently labeled with secondary antibodies (Dylight550-anti-rabbit-IgG, Thermo Fisher Scientific), and then stained with DAPI for detecting nuclei. Fluorescence images were obtained using an InCell Analyzer 2000 (GE Healthcare), and image analysis was done using Developer tool software.

## Results

### Time course of ERK* and Akt*

**Fig 2:**
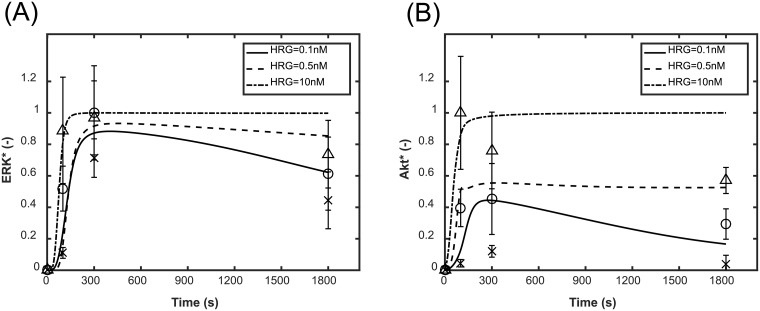
Time course simulation of ERK* and Akt*. (A) Simulated and experimental time course of ERK*. (B) Simulated and experimental time course of Akt*. The HRG concentrations were 0.1, 0.5 and 10 nM, while the EGF concentration was set to 0.5 nM. The solid, dashed and dot-dash lines indicate the simulated results at 0, 0.5 and 10 nM HRG, respectively. The cross, circle and triangle indicate the experimental activity at 0.1, 0.5 and 10 nM HRG, respectively. The initial activities are set to zero by subtracting the background intensity from the measured activities and then the resultant activities are normalized so that the maximum intensity during time course is 1. The error bars denote the standard deviations of signal intensities in quadruplicate independent experiments.

The kinetic model had been validated using the experimental response of ERK* and Akt* with respect to EGF and HRG stimulation in [11]. To further validate the model, we measured ERK* and Akt* in response to EGF and HRG using imaging cytometry, as shown in Fig 2. Both the simulated and experimental ERK* and Akt* increased initially and were then sustained, or decreased under all the conditions. Both the activities increased with an increase in HRG dose. These simulation results were relatively consistent with the experimental dynamics of ERK* and Akt*. Considering these dynamic behaviors of ERK* and Akt*, a high final activity value was consistent with high sustainability and long signaling duration. The final activities were employed to characterize sustained ERK* and Akt*.

**Fig 3:**
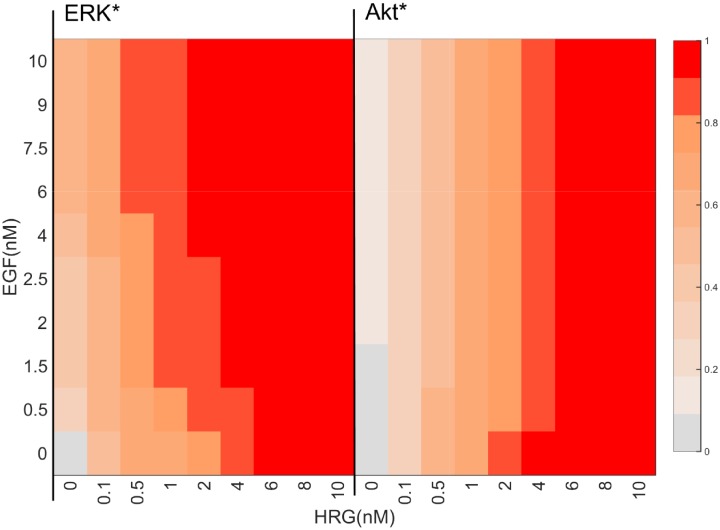
A heat map of the simulated final activity of ERK* and Akt*. The final ERK* and Akt* were simulated while systematically varying the ligand concentrations of EGF and HRG. The ERK* and Akt* were normalized by their maximum values. The red color becomes more intense with an increase in ERK* and Akt*. A solid red bar represents maximum activity.

To understand the input-output relationship, we simulated final ERK* and Akt*, while systematically changing the dose of EGF and HRG, as shown in Fig 3. The final ERK* was enhanced by HRG or EGF. ERK* increased steeply to half activity (0.5) even when stimulated with 0.1 nM HRG, and became saturated (>0.9) at more than 0.5 nM HRG. EGF stimulation increased ERK*, but did not induce a high final ERK* (>0.9) without HRG stimulation. Thus, the effect of EGF on ERK* was smaller than that of HRG. On the other hand, Akt* gradually increased to half activity at 0.5 nM HRG, and showed high activities (>0.9) above 4 nM HRG, while the response to EGF was minimal. Akt* showed a gradual, wide-range response to HRG, but a blunt response to EGF.

### Time course of DS for ERK* and Akt*

**Fig 4:**
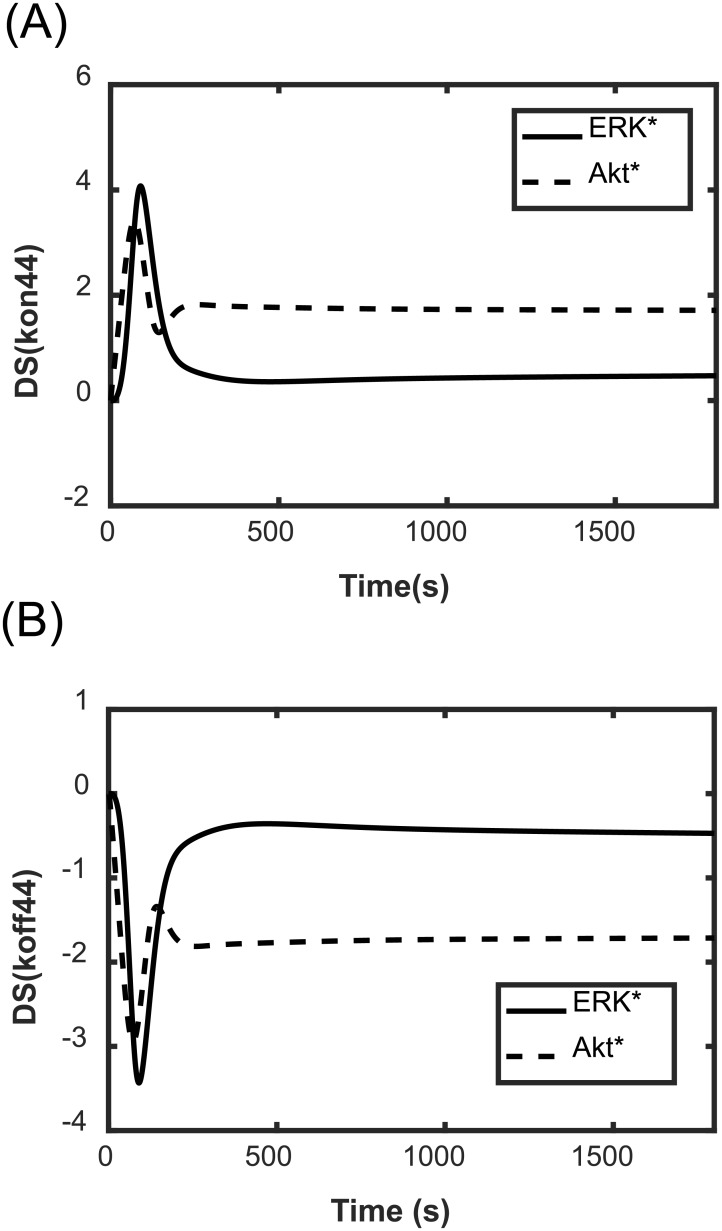
Time course of the DSs of ERK* and Akt*. The simulated DSs of ERK* and Akt* with respect to kon44 (A), and koff44 (B). The concentrations of EGF and HRG were 0.5 nM and 0.5 nM, respectively.

To illustrate how the reactions critically responsible for ERK* and Akt* change with time and in response to different doses of EGF and HRG, we simulated the DSs of ERK* and Akt* with respect to 237 kinetic parameters. Fig 4 shows the two representative patterns of the DS time course. These DSs reached maximum or minimum levels around 100 s and then decreased or increased, approaching a steady-state. The timing of local maximum or local minimum corresponded to the steepest gradients of ERK* and Akt* dynamics.

**Fig 5:**
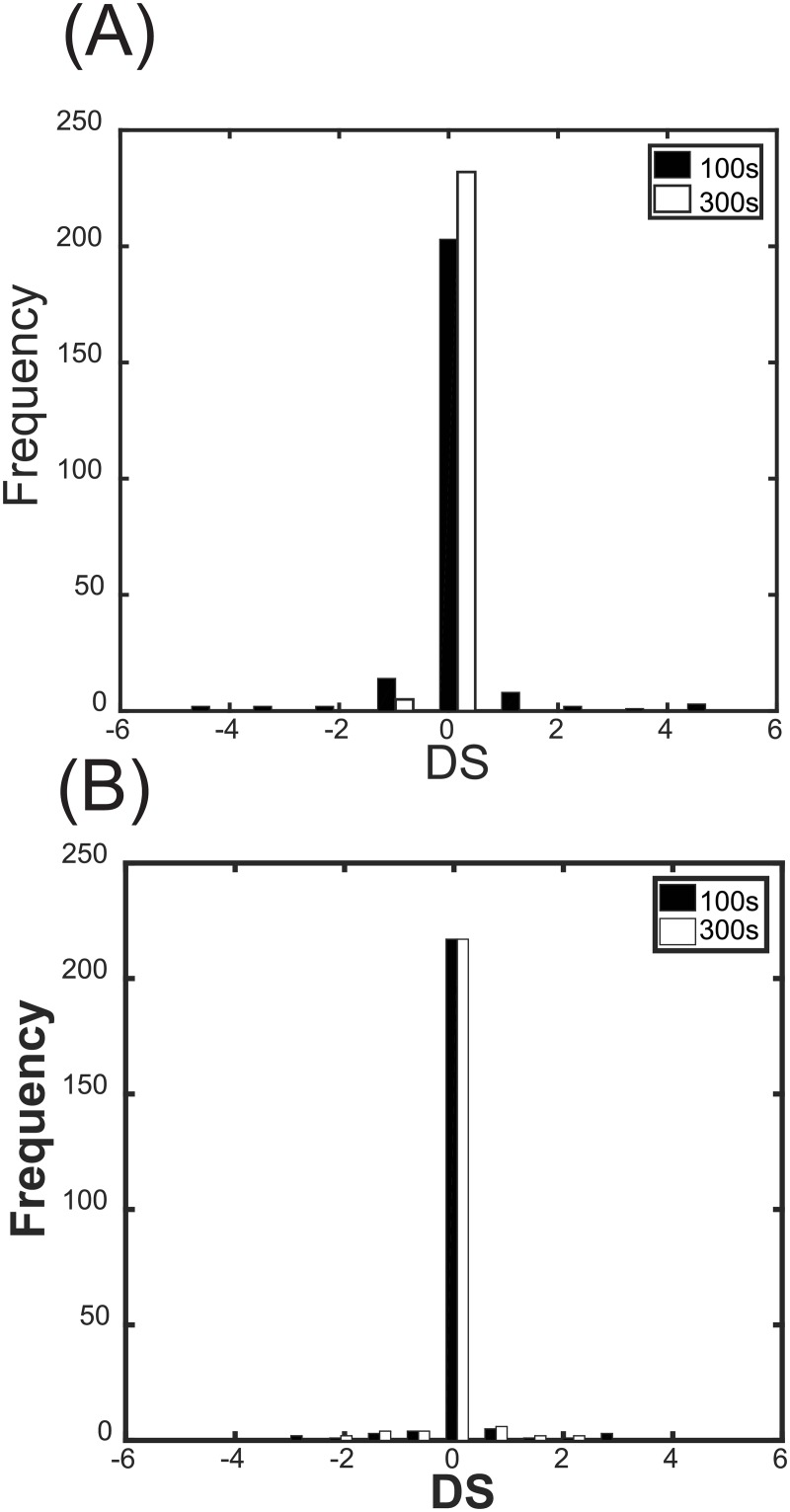
Frequency distributions of the DSs of ERK* and Akt*. (A) Frequency distribution of the DSs of ERK*. (B) Frequency distribution of the DSs of Akt*. DSs (n = 237) were simulated at 100 s and 300 s with (HRG, EGF) = (0.5 nM, 0.5 nM). The black and white bars indicate the distributions at 100 s and 300 s.

**Fig 6:**
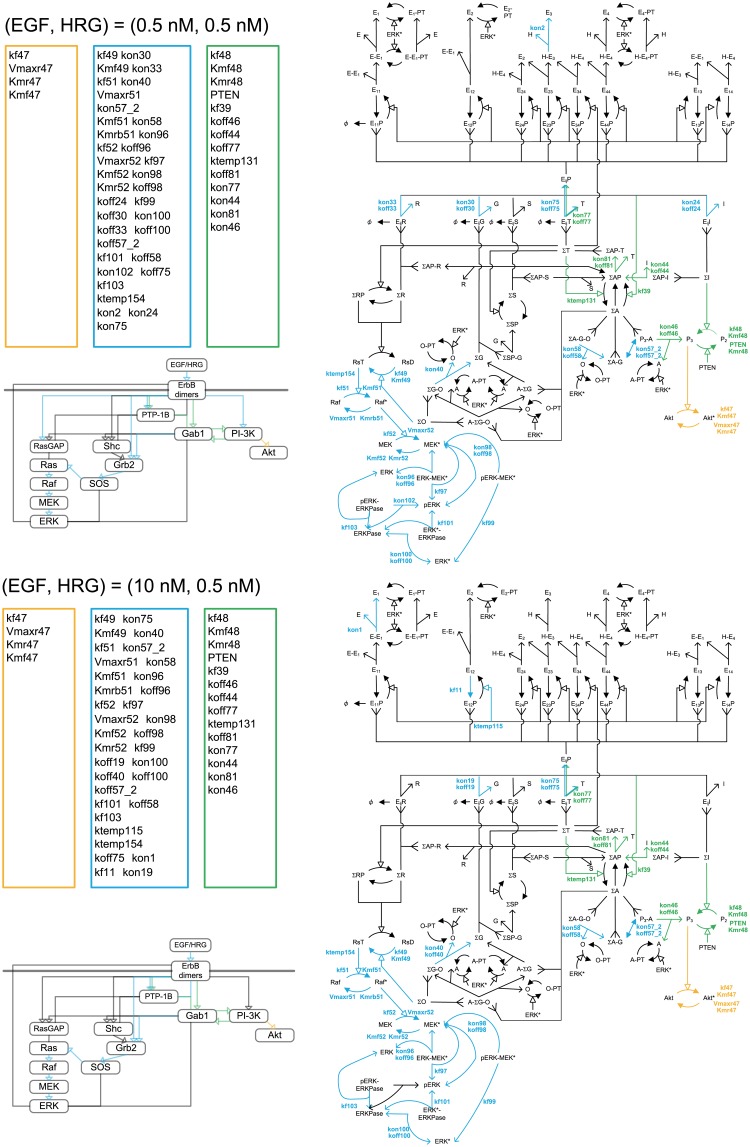
Critical parameter shifts in the early stage in response to EGF at a low HRG concentration. Orange, blue and green colors indicates the Akt*-specific, ERK*-specific, and dual-specific critical reactions or parameters at 100s with (EGF, HRG) = (0.5nM, 0.5nM) and (EGF, HRG) = (10.0nM, 0.5nM).

**Fig 7:**
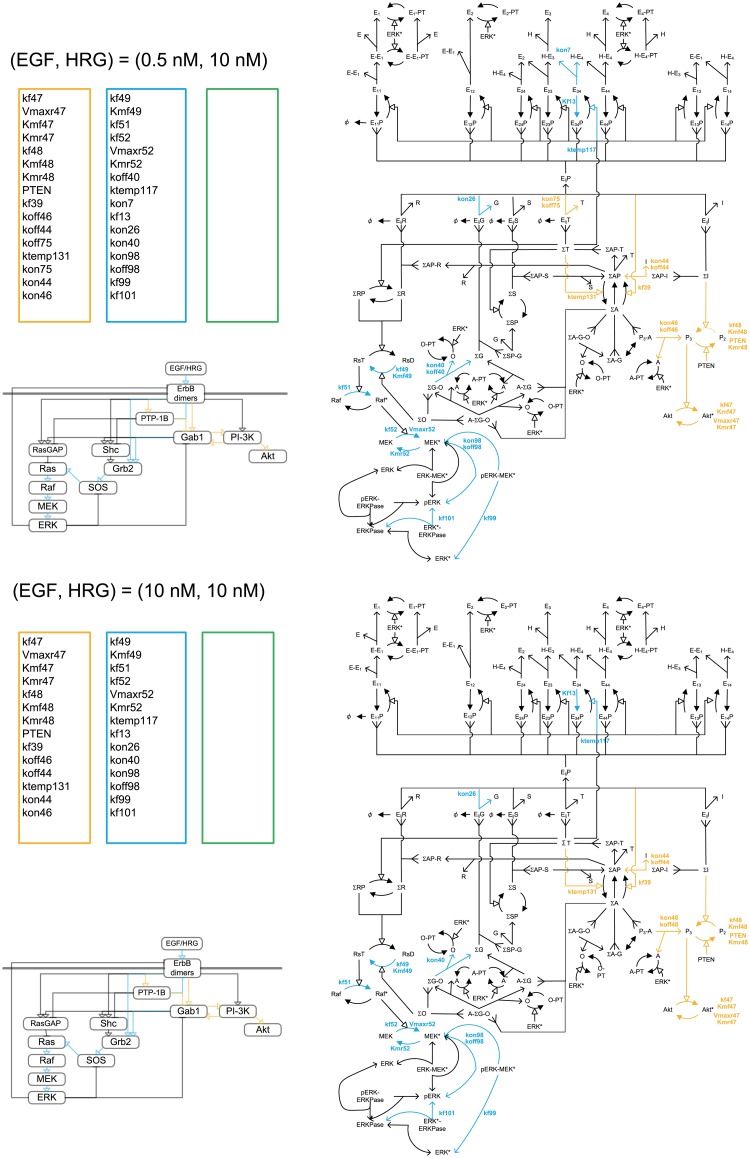
Critical parameter shifts in the early stage in response to EGF at a high HRG concentration. Orange, blue and green colors indicates the Akt*-specific, ERK*-specific, and dual-specific critical reactions or parameters at 100s with (EGF, HRG) = (0.5nM, 10.0nM) and (EGF, HRG) = (10.0nM, 10.0nM).

**Fig 8:**
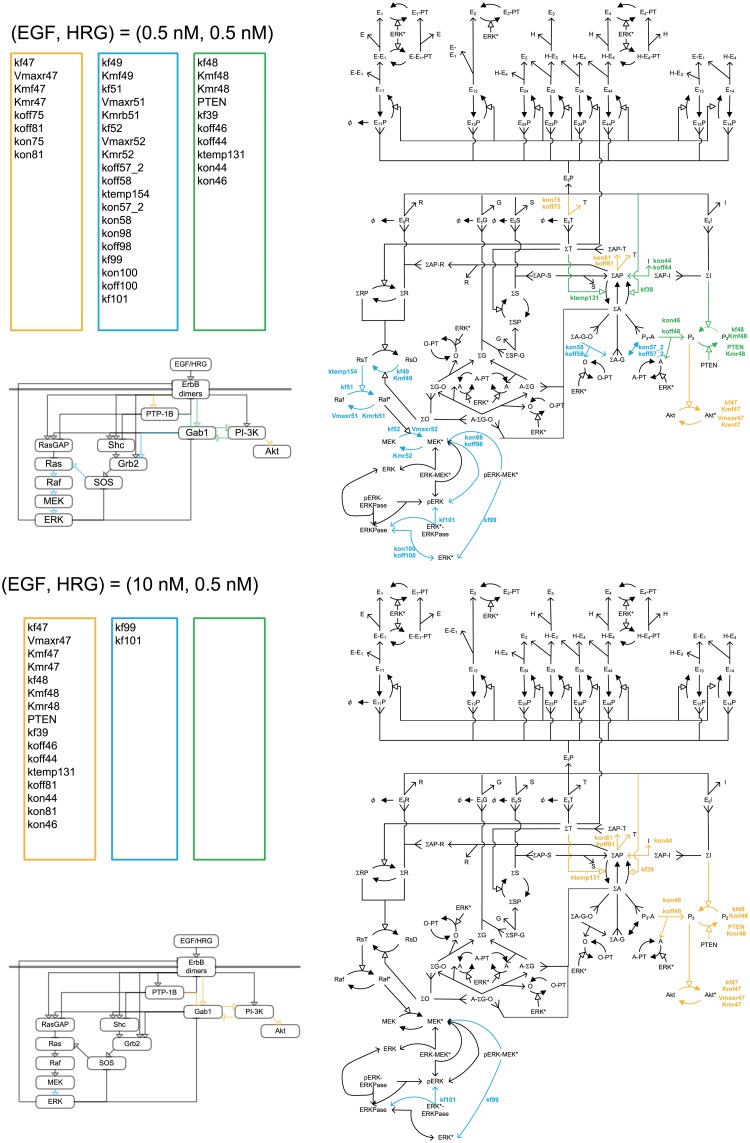
Critical parameter shifts in the late stage in response to EGF at a low HRG concentration. Orange, blue and green colors indicates the Akt*-specific, ERK*-specific, and dual-specific critical reactions or parameters at 300s with (EGF, HRG) = (0.5nM, 0.5nM) and (EGF, HRG) = (10.0nM, 0.5nM).

**Fig 9:**
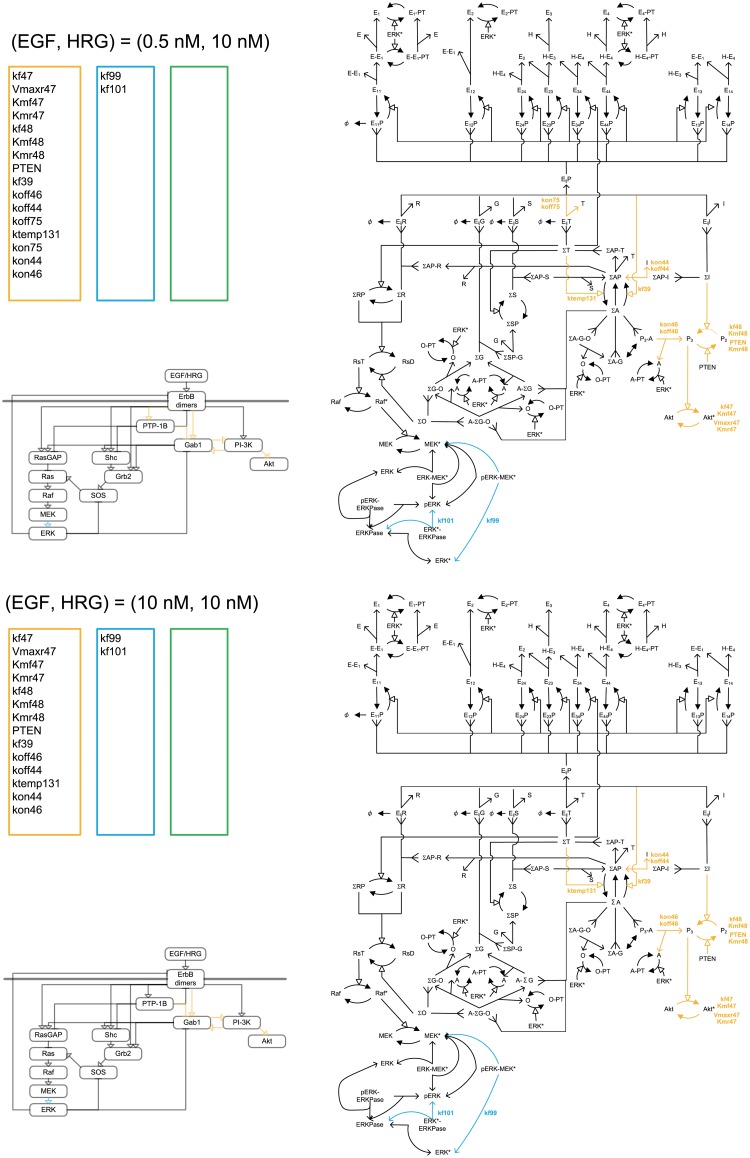
Critical parameter shifts in the late stage in response to EGF at a high HRG concentration. Orange, blue and green colors indicates the Akt*-specific, ERK*-specific, and dual-specific critical reactions or parameters at 300s with (EGF, HRG) = (0.5nM, 10.0nM) and (EGF, HRG) = (10.0nM, 10.0nM).

Fig 5 shows the distribution of 237 kinetic parameter DSs of ERK* and Akt* at 100 s, 300 s, and 1,800 s. The distributions were considerably more outlier-prone than the normal distribution. A limited number of the parameters had very high DSs for both ERK* and Akt*, while many parameters had very small DSs. Therefore, it appeared that a small number of reactions determine the dynamics of ERK* and Akt*. As shown in Figs 6–9, the critical parameters can be classified into three distinct groups: ERK*-specific highly sensitive parameters, Akt*-specific highly sensitive parameters, and both ERK*- and Akt*-specific highly sensitive parameters. Here, ERK* DS parameters of more than 0.25, and Akt* DS parameters of less than 0.55 were deemed ERK*-specific critical parameters. Similarly, ERK* DS parameters of less than 0.25, and Akt* DS parameters of more than 0.55 were deemed Akt*-specific critical parameters. Common critical parameters were defined as those with ERK* DSs of more than 0.25, and Akt* DSs of more than 0.55. Since we focused on the relative transitions in critical parameters with respect to time and ligand dose, the threshold values were determined so that they can intelligibly describe relative transitions.

In the early stages following activation with 0.5 nM HRG and 0.5 nM EGF, three classes of critical parameters were estimated (Figs 6 and 7). The critical reactions for ERK activation were distributed among multiple competitive pathways: ErbB dimers→→PTP-1B→Gab1→GrB2→SOS→Ras→Raf→MEK→ERK*, ErbB dimers→Grb2→SOS→Ras→Raf→MEK→ERK*, and ErbB dimers→RasGAP→Ras→Raf→MEK→ERK*. Interestingly, a few critical reactions were found on circuitous pathways that are distant from ERK* and close to Akt*: ErbB dimers→PI-3K, and ErbB dimers→Gab1↔ PI-3K. The PI-3K and Gab1 pathways to Akt* may compete with the other pathways to ERK*, because PI-3K and Gab1 are located at the branching points for ERK and Akt activation. The pathways of ErbB dimers→PI-3K →Akt* and ErbB dimers→PTP-1B→Gab1→Akt* were critical for Akt activation. The pathway of ErbB dimers→PTP-1B→Gab1↔PI-3K was common to activation of ERK and Akt. At 0.5 nM HRG and 10 nM EGF, the number of critical pathways for both ERK and Akt activation decreased. With an increase in HRG to 10.0 nM, the common critical reactions disappeared, then Akt*-specific pathways became dominant (Fig 7).

In the late stage (Figs 8 and 9), at 0.5 nM of HRG and 0.5 nM of EGF, three classes of critical parameters were estimated, and the number of ERK*-specific critical parameters decreased compared with the early stage. The critical pathways linked to ERK activation converged to the pathway: ErbB dimers→PTP-1B→Gab1→GrB2→SOS→Ras→Raf→MEK→ERK*. The pathway of ErbB dimers→PTP-1B and PI-3K→Akt* was critical for Akt activation. The pathway of ErbB dimers→PTP-1B→Gab1↔ PI-3K were the common critical reactions. As the EGF or HRG doses increased, the common critical parameters disappeared, then Akt*-specific critical parameters became dominant. An increase in EGF or HRG dose made the critical parameter distributions converge to almost the same distribution. The critical reactions for ERK* were located just upstream of ERK*; those for Akt* were in the pathway of ErbB dimers→PTP-1B→Gab1↔ PI-3K→Akt.

### MPS dynamics

**Fig 10:**
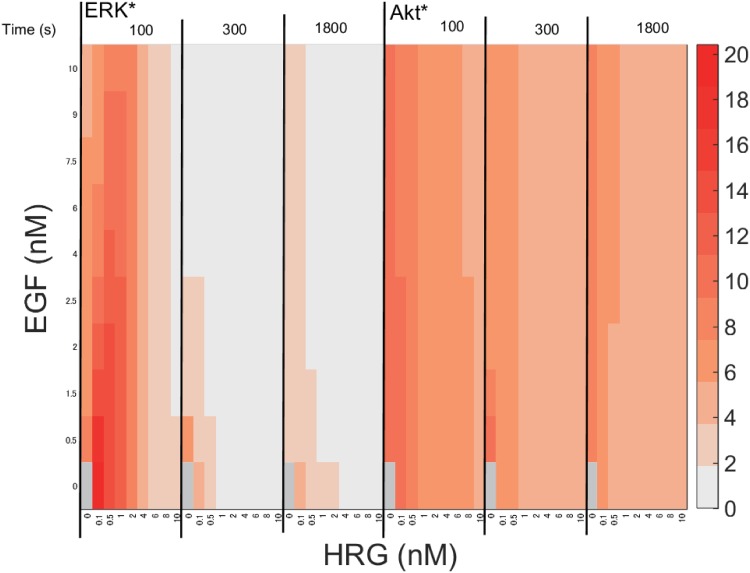
MPS time course of ERK* and Akt* in response to different concentrations of EGF and HRG. The heat map illustrates the MPS values of ERK* and Akt* simulated at different concentrations of EGF and HRG. The white color represents a very small MPS value, i.e., enhanced robustness with respect to external changes in EGF and HRG, while the red color represents a high MPS value, i.e., sensitivity with respect to external changes in EGF and HRG.

Single parameter sensitivity is effective for exploring a specific reaction that is sensitive to perturbation, but it cannot characterize the robustness of the entire system. Generally, some single sensitivities increase and others decrease, thus it is difficult to precisely estimate the robustness of the entire system. We calculated the MPSs of ERK* and Akt* with respect to all kinetic parameters to estimate the robustness of ERK* and Akt* to perturbations to intracellular kinetics (Fig 10). The MPSs of both ERK* and Akt* remarkably decreased after 100 s, and they also decreased concomitant with an increase in EGF or HRG. The MPSs were more suppressed by HRG than by EGF. The MPSs of ERK* were considerably less than those of Akt* at each time and each dose, indicating that ERK* is more robust than Akt* to perturbations to intracellular kinetics.

### Effect of inhibitors

**Fig 11:**
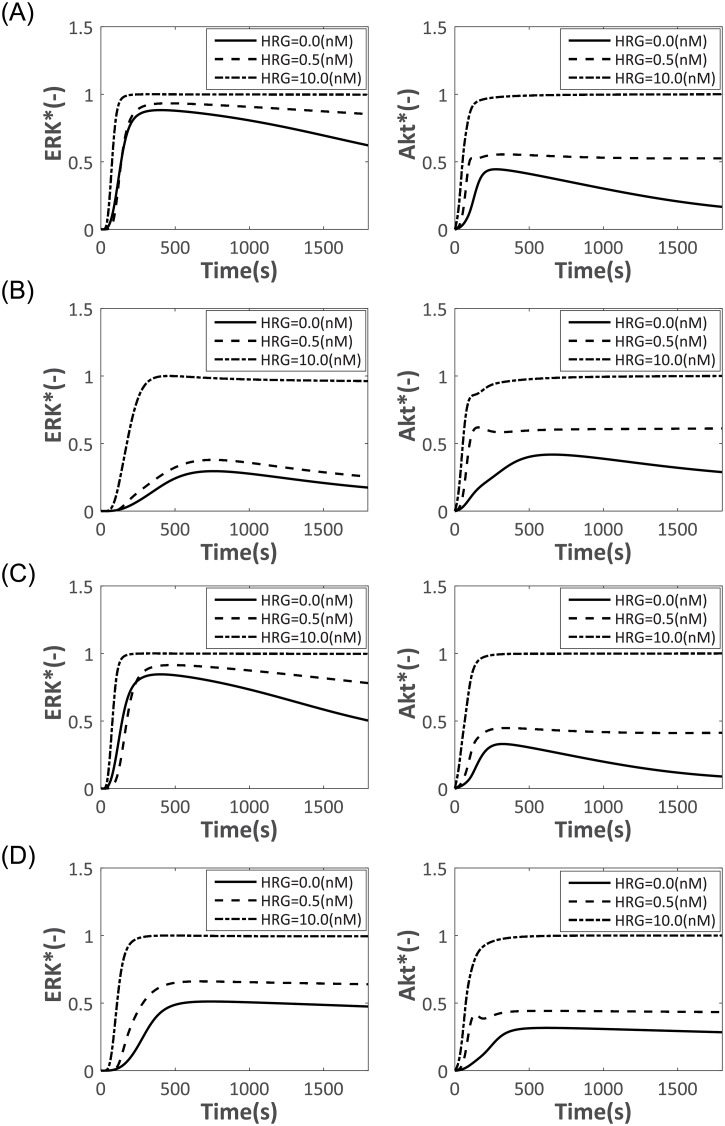
Dynamics of ERK* and Akt* in response to the addition of three inhibitors. (A) Control. No inhibitor added. (B) ERK inhibitor (9.0 nM). (C) Akt inhibitor (0.6 nM). (D) ErbB inhibitor (30 nM). The inhibitors were added at 0 s. The black lines, dashed lines and dotted lines indicate the simulated time courses of ERK* and Akt* at HRG concentrations of 0 nM, 0.5 nM and 10.0 nM, respectively. The EGF concentration was fixed at 10.0 nM.

To further investigate network architectures, we performed pathway inhibitor experiments *in silico*. Three inhibitors (a MEK inhibitor, an Akt inhibitor, and an ErbB inhibitor) were added to the signaling network (Fig 11). Addition of the inhibitors can be regarded as intracellular perturbations, because the inhibitors change the kinetic parameters of their associated proteins. The MEK inhibitor delayed the start-up of both ERK* and Akt* and suppressed pathway activation. The ERK pathway inhibitor suppressed HRG- or EGF-induced ERK* [11]. The Akt inhibitor specifically suppressed Akt*, with minimal effect on ERK*, suggesting that Akt activation does not affect the ERK activation pathways, despite the extensive crosstalk between the two. The ErbB inhibitor, which blocks ligand binding to the receptor, delayed the start-up of both ERK* and Akt* and suppressed pathway activation. Since the ErbB receptor is upstream of ERK* and Akt*, both pathways were affected. It is difficult to control ERK* with these inhibitors because ERK pathways connect upstream of Akt* or through feedback loops to ErbB dimers and Gab1.

## Discussion

### Systematic analysis

To demonstrate the feasibility of the developed DS simulator, we analyzed the robustness of the ERK* and Akt* with respect to a combination of EGF and HRG. In general, it is difficult to systematically test many ligand or drug combinations *in vivo*, because clinical testing is expensive and time-consuming. Systematic analysis of kinetic models of ErbB signaling can overcome such problems, because models can computationally predict the output properties for combinations of multiple ligands. A major challenge for ErbB signaling studies is to understand how a combination of stimuli gives rise to different responses despite the promiscuous activation of shared pathways. Birtwistle et al. (2007) [11] built a detailed kinetic model of the ErbB signaling network and performed perturbation analysis to reveal how the network generates robust ERK and Akt activation, but the model was not amenable to a comprehensive mathematical analysis. We present a systematic analysis for understanding how a response to the two ligands alters the output properties of ERK* and Akt*, and how the network generates a robust response to different types of perturbations. To characterize the output response of ERK* and Akt*, the final activities were used. Many signaling pathways that transmit information from ErbB dimerization to ERK or Akt activation are not direct, and there is extensive overlap and crosstalk, thus it is difficult to discriminate between critical pathways and those that are less important. We used single parameter sensitivity to predict critical reactions in the many possible signaling pathways. Reactions with a low sensitivity indicated that they were saturated or their contributions were smaller than those of competitive or alternative pathways. In branching cascades, the reaction pathways generated from one molecular species can compete with each other, and an increase in one reaction causes others to decrease. MPS was employed to characterize the robustness of ERK* and Akt* to perturbations to all intracellular kinetics.

In addition to system analysis, we developed the DDM-based DS simulator that solves the exact value of the DS without approximation, while the IDMs use approximate sensitivity values [22, 23]. In principle, the accuracy of DDM outperforms IDM.

### Robustness and response

We focused on the DSs of ERK* and Akt* in the ErbB signaling network of MCF-7 cells because the duration of activity plays an important role in the control of proliferation, survival and differentiation in response to EGF and HRG. ERK* increased steeply with the increase in HRG and was saturated at a low dose of HRG, while showing a gently rising response to EGF. Akt* had a gradual wide-range response to HRG dose, but the response to the change in EGF was minimal. We predicted the switching or output properties of ERK* and Akt* in response to the combination of EGF and HRG. As far as robustness is concerned, the ERK* was robust to intracellular perturbations, because ERK* is controlled by multiple negative feedback loops (e.g., ERK*→Gab1→RasGAP→Ras→Raf→MEK→ERK*, ERK*→Gab1→Grb2→SOS→Ras→Raf→MEK→ERK*, ERK*→Gab1→Shc→Grb2→SOS→Ras→Raf→MEK→*ERK, ERK*→SOS→Ras→Raf→MEK→ERK*, ERK*→ErbB_dimers→RasGAP→Ras→Raf→MEK→ERK*) [24]. On the other hand, Akt* was sensitive to perturbations in intracellular kinetics. Note that Akt* is not regulated by negative feedback loops. Inhibitor addition suggested that those feedback loops play a significant role in connecting ERK* to Akt* through ErbB dimers, PI-3K, or Gab1, because the ERK* specific inhibitor affected not only ERK*, but also Akt*.

### Critical reactions responsible for ERK* and Akt*

DS analysis of 237 kinetic parameters involved in ERK* and Akt* predicted that only a small number of parameters were critical and governed the robustness of the model. Although frequent crosstalk is found in mammalian signaling pathways, a fundamental question is which of these pathways are critical or dominant in response to different conditions and stimuli. While network or pathway maps cannot address this issue due to a lack of quantitative information, sensitivity analysis can facilitate the identification of critical pathways. We estimated that a set of critical parameters changed in response to ligand doses and time. The critical parameters were widely distributed at the early time points or at low doses of EGF and HRG, suggesting that many pathways were involved in signal transduction. The reaction pathway from ErbB dimers to PI-3K to Akt* was responsible for ERK activation at low doses of HRG and EGF. This is because the PI-3K pathways compete with pathways activated by ErbB dimerization to drive Akt activation, or signaling downstream of PI-3K is connected to ERK activation through circuitous pathways. Since the pathways from ErbB dimers to PTP-1B and Gab1 are common to ERK and Akt activation, it is reasonable to assume that that they play a dominant role in ERK and Akt activation. As EGF and HRG dose increased over time there was a dynamic transition of critical reactions, and the critical parameters common to ERK* and Akt* were Akt*-specific.

## Conclusion

Using our DDM-based DS simulator, we estimated the response characteristics and robustness of the ErbB signaling network in cancer cells. The data showed how the reactions critically responsible for ERK* and Akt* changed with time and in response to different doses of EGF and HRG, and illustrated that only a small number of reactions determine ERK* and Akt*. Akt* was sensitive to perturbations in intracellular kinetics, while ERK* was more robust due to multiple, negative feedback loops. ERK* increased steeply concomitant with an increase in HRG dose until saturation, while showing a gently rising response to EGF. Akt* had a gradual wide-range response to HRG, and a blunt response to EGF.

## Supporting information

S1 Table Differential equations. (PDF)

S2 Table Reaction rate equations. (PDF)

S3 Table Fraction multipliers for seeding molecular species dissociation. (PDF)

S4 Table Molecular species. (PDF)

S5 Table Kinetic parameter list. (PDF)

S6 Table Initial concentration of molecular species. (PDF)
