# Supplementary material for: Robustness analysis of the detailed kinetic model of an ErbB signaling network by using dynamic sensitivity
Source: PLoS One. 2017 May 24;12(5):e0178250. doi: 10.1371/journal.pone.0178250 (PMC5443533; doi:10.1371/journal.pone.0178250)
Supplement: S1 Table — (PDF) [file pone.0178250.s001.pdf]

Table S1 Differential equations

Differential equations
$\frac{d}{dt}(E)=$ -R1-R86
$\frac{d}{dt}(H)=$ -R2-R3-R87
$\frac{d}{dt}(E_1)=$ -R1*VeVc-R81
$\frac{d}{dt}(E_2)=$ -R5-R6-R8-R82
$\frac{d}{dt}(E_3)=$ -R2*VeVc
$\frac{d}{dt}(E_4)=$ -R3*VeVc-R83
$\frac{d}{dt}(E-E_1)=$ R1*VeVc-2*R4-R5-R56-R57-R84
$\frac{d}{dt}(H-E_3)=$ R2*VeVc-R6-R7-R56
$\frac{d}{dt}(H-E_4)=$ R3*VeVc-R7-R8-2*R9-R57-R85
$\frac{d}{dt}(E_{11})=$ R4-R10
$\frac{d}{dt}(E_{12})=$ R5-R11
$\frac{d}{dt}(E_{23})=$ R6-R12
$\frac{d}{dt}(E_{34})=$ R7-R13
$\frac{d}{dt}(E_{24})=$ R8-R14
$\frac{d}{dt}(E_{44})=$ R9-R15
$\frac{d}{dt}(E_{11}P)=$ R10-R16-R17-R18-R73
$\frac{d}{dt}(E_{12}P)=$ R11-R19-R20-R21-R74
$\frac{d}{dt}(E_{23}P)=$ R12-R22-R23-R24-R25-R75
$\frac{d}{dt}(E_{34}P)=$ R13-R26-R27-R28-R29-R76
$\frac{d}{dt}(E_{24}P)=$ R14-R30-R31-R32-R33-R77
$\frac{d}{dt}(E_{44}P)=$ R15-R35-R36-R37-R78-R34
$\frac{d}{dt}(G)=$ -R16-R19-R22-R26-R30-R34-R42-R60-R64-R105

$\frac{d}{dt}(S) =$ -R17-R20-R23-R27-R31-R35-R43-R61-R65
$\frac{d}{dt}(I) =$ -R24-R28-R32-R36-R62-R66-R44
$\frac{d}{dt}(R) =$ -R18-R21-R25-R29-R33-R37-R45-R63-R67
$\frac{d}{dt}(O) =$ -R40-R54-R68-R106
$\frac{d}{dt}(A) =$ -R41-R46-R55-R107
$\frac{d}{dt}(E_{11}G) =$ R16
$\frac{d}{dt}(E_{11}S) =$ R17
$\frac{d}{dt}(E_{11}R) =$ R18
$\frac{d}{dt}(E_{12}G) =$ R19
$\frac{d}{dt}(E_{12}S) =$ R20
$\frac{d}{dt}(E_{12}R) =$ R21
$\frac{d}{dt}(E_{23}G) =$ R22
$\frac{d}{dt}(E_{23}S) =$ R23
$\frac{d}{dt}(E_{23}I) =$ R24
$\frac{d}{dt}(E_{23}R) =$ R25
$\frac{d}{dt}(E_{34}G) =$ R26
$\frac{d}{dt}(E_{34}S) =$ R27
$\frac{d}{dt}(E_{34}I) =$ R28
$\frac{d}{dt}(E_{34}R) =$ R29
$\frac{d}{dt}(E_{24}G) =$ R30
$\frac{d}{dt}(E_{24}S) =$ R31
$\frac{d}{dt}(E_{24}I) =$ R32
$\frac{d}{dt}(E_{24}R) =$ R33
$\frac{d}{dt}(E_{44}G) =$ R34

$\frac{d}{dt}(E_{44}S) =$ R35
$\frac{d}{dt}(E_{44}I) =$ R36
$\frac{d}{dt}(E_{44}R) =$ R37
$\frac{d}{dt}(\sum G) =$ -R40-R41+R16+R19+R22+R26+R30+R34+R42+R60+R64
$\frac{d}{dt}(\sum S) =$ -R38+R17+R20+R23+R27+R31+R35+R43+R61+R65
$\frac{d}{dt}(\sum I) =$ R24+R28+R32+R36+R44+R62+R66
$\frac{d}{dt}(\sum R) =$ R18+R21+R25+R29+R33+R37+R45-R50+R63+R67
$\frac{d}{dt}(\sum A) =$ -R39+R41+R46+R107
$\frac{d}{dt}(\sum SP) =$ R38-R42
$\frac{d}{dt}(\sum AP) =$ R39-R43-R44-R45-R108
$\frac{d}{dt}(\sum G-O) =$ R40-R107
$\frac{d}{dt}(\sum G-A) =$ R41-R68
$\frac{d}{dt}(\sum SP-G) =$ R42
$\frac{d}{dt}(\sum AP-S) =$ R43
$\frac{d}{dt}(\sum AP-I) =$ R44
$\frac{d}{dt}(\sum AP-R) =$ R45
$\frac{d}{dt}(P_3-A) =$ R46-R105
$\frac{d}{dt}(P_2) =$ -R48
$\frac{d}{dt}(P_3) =$ -R46+R48
$\frac{d}{dt}(Akt) =$ -R47
$\frac{d}{dt}(Akt*) =$ R47
$\frac{d}{dt}(RsD) =$ -R49
$\frac{d}{dt}(RsT) =$ R49
$\frac{d}{dt}(\sum RP) =$ R50

$\frac{d}{dt}\left(Raf\right)=$ -R51
$\frac{d}{dt}\left(Raf\ast\right)=$ R51
$\frac{d}{dt}\left(MEK\right)=$ -R52
$\frac{d}{dt}\left(MEK\ast\right)=$ R52-R96+R97-R98+R99
$\frac{d}{dt}\left(ERK\right)=$ -R96+R103
$\frac{d}{dt}\left(ERK\ast\right)=$ R99-R100
$\frac{d}{dt}\left(OP\right)=$ R54
$\frac{d}{dt}\left(AP\right)=$ R55
$\frac{d}{dt}\left(A-\sum G-O\right)=$ R68+R107
$\frac{d}{dt}\left(\sum A-G\right)=$ R105-R106
$\frac{d}{dt}\left(\sum A-G-O\right)=$ R106
$\frac{d}{dt}\left(\sum O\right)=$ R40+R68+R106
$\frac{d}{dt}\left(E_{13}\right)=$ R56-R58
$\frac{d}{dt}\left(E_{14}\right)=$ R57-R59
$\frac{d}{dt}\left(E_{13}P\right)=$ R58-R60-R61-R62-R63-R79
$\frac{d}{dt}\left(E_{14}P\right)=$ R59-R64-R65-R66-R67-R80
$\frac{d}{dt}\left(E_{13}G\right)=$ R60
$\frac{d}{dt}\left(E_{13}S\right)=$ R61
$\frac{d}{dt}\left(E_{13}I\right)=$ R62
$\frac{d}{dt}\left(E_{13}R\right)=$ R63
$\frac{d}{dt}\left(E_{14}G\right)=$ R64
$\frac{d}{dt}\left(E_{14}S\right)=$ R65
$\frac{d}{dt}\left(E_{14}I\right)=$ R66
$\frac{d}{dt}\left(E_{14}R\right)=$ R67

$\frac{d}{dt}\left(f_{\text{int}} \right) =$ R104
$\frac{d}{dt}\left(T \right) =$ -R73-R74-R75-R76-R77-R78-R79-R80-R108
$\frac{d}{dt}\left(E_{11}T \right) =$ R73
$\frac{d}{dt}\left(E_{12}T \right) =$ R74
$\frac{d}{dt}\left(E_{23}T \right) =$ R75
$\frac{d}{dt}\left(E_{34}T \right) =$ R76
$\frac{d}{dt}\left(E_{24}T \right) =$ R77
$\frac{d}{dt}\left(E_{44}T \right) =$ R78
$\frac{d}{dt}\left(E_{13}T \right) =$ R79
$\frac{d}{dt}\left(E_{14}T \right) =$ R80
$\frac{d}{dt}\left(\sum AP - T \right) =$ R108
$\frac{d}{dt}\left(\sum T \right) =$ R73+R74+R75+R76+R77+R78+R79+R80+R108
$\frac{d}{dt}\left(E_1 - PT \right) =$ -R86*VeVc+R81
$\frac{d}{dt}\left(E_2 - PT \right) =$ R82
$\frac{d}{dt}\left(E_4 - PT \right) =$ -R87*VeVc+R83
$\frac{d}{dt}\left(E - E_1 - PT \right) =$ R86*VeVc+R84
$\frac{d}{dt}\left(H - E_4 - PT \right) =$ R87*VeVc+R85
$\frac{d}{dt}\left(pERK \right) = 0$
$\frac{d}{dt}\left(ERK - MEK* \right) = 0$
$\frac{d}{dt}\left(pERK - MEK* \right) = 0$
$\frac{d}{dt}\left(ERKPase \right) = 0$
$\frac{d}{dt}\left(ERK* - ERKPase \right) = 0$
$\frac{d}{dt}\left(pERK - ERKPase \right) = 0$
$\frac{d}{dt}\left(xconc121 \right) =$ R96-R97

$\frac{d}{dt}\left(xconc122\right)=$ R97-R98+R101-R102
$\frac{d}{dt}\left(xconc123\right)=$ R98-R99
$\frac{d}{dt}\left(xconc124\right)=$ -R100+R101-R102+R103
$\frac{d}{dt}\left(xconc125\right)=$ R100-R101
$\frac{d}{dt}\left(xconc126\right)=$ R102-R103

VeVc is taken to be 33.3 [Kholodenko et al., 1999]. Refer to TableS1~S6 for the details of the species and reactions.
