# Supplementary material for: Robustness analysis of the detailed kinetic model of an ErbB signaling network by using dynamic sensitivity
Source: PLoS One. 2017 May 24;12(5):e0178250. doi: 10.1371/journal.pone.0178250 (PMC5443533; doi:10.1371/journal.pone.0178250)
Supplement: S2 Table — (PDF) [file pone.0178250.s002.pdf]

Table S2 Reaction rate equations

Index	*Description*	*Rate Equation*
R1	EGF binding to ErbB1	$k_{on1}[E][E_1]/VeVc - k_{off1}[E-E_1]/VeVc$
R2	HRG binding to ErbB3	$k_{on2}[H][E_3]/VeVc - k_{off2}[H-E_3]/VeVc$
R3	HRG binding to ErbB4	$k_{on3}[H][E_4]/VeVc - k_{off3}[H-E_4]/VeVc$
R4	ErbB1 homodimerization	$k_{on4}[E-E_1][E-E_1] - k_{off4}[E_{11}]$
R5	ErbB1/ErbB2 dimerization	$k_{on5}[E_2][E-E_1] - k_{off5}[E_{12}]$
R6	ErbB2/ErbB3 dimerization	$k_{on6}[E_2][H-E_3] - k_{off6}[E_{23}]$
R7	ErbB3/ErbB4 dimerization	$k_{on7}[H-E_4][H-E_3] - k_{off7}[E_{34}]$
R8	ErbB2/ErbB4 dimerization	$k_{on8}[H-E_4][E_2] - k_{off8}[E_{24}]$
R9	ErbB4 homodimerization	$k_{on9}[H-E_4][H-E_4] - k_{off9}[E_{44}]$
R10	1-1 phosphorylation	$\frac{k_{f10}[E_{11}]}{1+InH_3/KmI_3} - \frac{V_{\max10}[E_{11}P]}{K_{m10}+[E_{11}P]} - k_{temp114}[\sum T][E_{11}P]$
R11	1-2 phosphorylation	$\frac{k_{f11}[E_{12}]}{1+InH_3/KmI_3} - \frac{V_{\max11}[E_{12}P]}{K_{m11}+[E_{12}P]} - k_{temp115}[\sum T][E_{12}P]$
R12	2-3 phosphorylation	$\frac{k_{f12}[E_{23}]}{1+InH_3/KmI_3} - \frac{V_{\max12}[E_{23}P]}{K_{m12}+[E_{23}P]} - k_{temp116}[\sum T][E_{23}P]$
R13	3-4 phosphorylation	$\frac{k_{f13}[E_{34}]}{1+InH_3/KmI_3} - \frac{V_{\max13}[E_{34}P]}{K_{m13}+[E_{34}P]} - k_{temp117}[\sum T][E_{34}P]$
R14	2-4 phosphorylation	$\frac{k_{f14}[E_{24}]}{1+InH_3/KmI_3} - \frac{V_{\max14}[E_{24}P]}{K_{m14}+[E_{24}P]} - k_{temp118}[\sum T][E_{24}P]$
R15	4-4 phosphorylation	$\frac{k_{f15}[E_{44}]}{1+InH_3/KmI_3} - \frac{V_{\max15}[E_{44}P]}{K_{m15}+[E_{44}P]} - k_{temp119}[\sum T][E_{44}P]$

R16	Grb2 binding to E11	$4 * k_{on16}[G][E_{11}P]-k_{off16}[E_{11}G]\,f_{\sum G}$
R17	Shc binding to E11	$8 * k_{on17}[S][E_{11}P]-k_{off17}[E_{11}S]\,f_{\sum S}$
R18	RasGAP binding to E11	$2 * k_{on18}[R][E_{11}P]-k_{off18}[E_{11}R]\,f_{\sum R}$
R19	Grb2 binding to E12	$3 * k_{on19}[G][E_{12}P]-k_{off19}[E_{12}G]\,f_{\sum G}$
R20	Shc binding to E12	$6 * k_{on20}[S][E_{12}P]-k_{off20}[E_{12}S]\,f_{\sum S}$
R21	RasGAP binding to E12	$2 * k_{on21}[R][E_{12}P]-k_{off21}[E_{12}R]\,f_{\sum R}$
R22	Grb2 binding to E23	$3 * k_{on22}[G][E_{23}P]-k_{off22}[E_{23}G]\,f_{\sum G}$
R23	Shc binding to E23	$3 * k_{on23}[S][E_{23}P]-k_{off23}[E_{23}S]\,f_{\sum S}$
R24	PI-3K binding to E23	$3 * k_{on24}[I][E_{23}P]-k_{off24}[E_{23}I]$
R25	RasGAP binding to E23	$2 * k_{on25}[R][E_{23}P]-k_{off25}[E_{23}R]\,f_{\sum R}$
R26	Grb2 binding to E34	$4 * k_{on26}[G][E_{34}P]-k_{off26}[E_{34}G]\,f_{\sum G}$
R27	Shc binding to E34	$3 * k_{on27}[S][E_{34}P]-k_{off27}[E_{34}S]\,f_{\sum S}$
R28	PI-3K binding to E34	$4 * k_{on28}[I][E_{34}P]-k_{off28}[E_{34}I]$
R29	RasGAP binding to E34	$2 * k_{on29}[R][E_{34}P]-k_{off29}[E_{34}R]\,f_{\sum R}$
R30	Grb2 binding to E24	$3 * k_{on30}[G][E_{24}P]-k_{off30}[E_{24}G]\,f_{\sum G}$
R31	Shc binding to E24	$4 * k_{on31}[S][E_{24}P]-k_{off31}[E_{24}S]\,f_{\sum S}$
R32	PI-3K binding to E24	$1 * k_{on32}[I][E_{24}P]-k_{off32}[E_{24}I]$
R33	RasGAP binding to E24	$2 * k_{on33}[R][E_{24}P]-k_{off33}[E_{24}R]\,f_{\sum R}$
R34	Grb2 binding to E44	$4 * k_{on34}[G][E_{44}P]-k_{off34}[E_{44}G]\,f_{\sum G}$

R35	Shc binding to E44	$4*k_{on35}[S][E_{44}P]-k_{off35}[E_{44}S]\,f_{\sum S}$
R36	PI-3K binding to E44	$2*k_{on36}[I][E_{44}P]-k_{off36}[E_{44}I]$
R37	RasGAP binding to E44	$2*k_{on37}[R][E_{44}P]-k_{off37}[E_{44}R]\,f_{\sum R}$
R38	Membrane localized Shc phosphorylation	$k_{f38}\left[\sum S\right]\left[\sum EP\right]-\frac{V_{\max 38}\left[\sum SP\right]}{K_{m38}+\left[\sum SP\right]}-k_{temp130}\left[\sum T\right]\left[\sum SP\right]$
R39	Membrane localized Gab1 phosphorylation	$k_{f39}\left[\sum A\right]\left[\sum EP\right]-\frac{V_{\max 39}\left[\sum AP\right]}{K_{m39}+\left[\sum AP\right]}-k_{temp131}\left[\sum T\right]\left[\sum AP\right]$
R40	SOS binding to membrane localized Grb2	$k_{on40}\left[\sum G\right][O]-k_{off40}\left[\sum G-O\right]$
R41	Gab1 binding to membrane localized Grb2	$k_{on41}\left[\sum G\right][A]-k_{off41}\left[\sum G-A\right]f_{\sum A}$
R42	Grb2 binding to membrane localized Shc-P	$k_{on42}\left[\sum SP\right][G]-k_{off42}\left[\sum SP-G\right]f_{\sum G}$
R43	Shc binding to membrane localized Gab1-P	$3*k_{on43}\left[\sum AP\right][S]-k_{off43}\left[\sum AP-S\right]f_{\sum S}$
R44	PI-3K binding to membrane localized Gab1-P	$3*k_{on44}\left[\sum AP\right][I]-k_{off44}\left[\sum AP-I\right]$
R45	RasGAP binding to membrane localized Gab1-P	$2*k_{on45}\left[\sum AP\right][R]-k_{off45}\left[\sum AP-R\right]f_{\sum R}$
R46	Gab1 binding to PIP3	$k_{on46}[P_3][A]-k_{off46}[P_3-A]\,f_{\sum A}$
R47	Akt to Akt*	$\frac{k_{f47}[P_3][Akt]}{K_{mf47}+[Akt]}-\frac{V_{\max r47}[Akt*]}{K_{mr47}+[Akt*]}$
R48	PIP2 to PIP3	$\frac{k_{f48}(1-f_{\text{int}}*f_{11})\left[\sum I\right][P_2]}{K_{mf48}*(1+InH_2/KmI_2)+[P_2]}-\frac{k_{r48}[PTEN][P_3]}{K_{mr48}+[P_3]},\quad f_{11}=\frac{[E_{11}P]}{\left[\sum EP\right]}$

R49	RasGDP to RasGTP	$\frac{k_{f49}\left[\sum O\right]\left[RsD\right]}{K_{mf49}+\left[RsD\right]}-\frac{k_{r49}\left[\sum R\right]\left[RsT\right]}{K_{mr49}+\left[RsT\right]}-\frac{k_{r49b}\left[\sum RP\right]\left[RsT\right]}{K_{mr49b}+\left[RsT\right]}-k_{temp154}\left[RsT\right]$
R50	Membrane localized RasGAP phosphorylation	$k_{f50}\left[\sum R\right]\left[\sum EP\right]-\frac{V_{\max 50}\left[\sum RP\right]}{K_{m50}+\left[\sum RP\right]}-k_{temp133}\left[\sum T\right]\left[\sum RP\right]$ $\sum EP=\left[E_{11}P\right]+\left[E_{12}P\right]+\left[E_{23}P\right]+\left[E_{24}P\right]+\left[E_{34}P\right]+\left[E_{44}P\right]+\left[E_{13}P\right]+\left[E_{14}P\right]$
R51	Raf to Raf*	$\frac{k_{f51}\left[RsT\right]\left[Raf\right]}{K_{mf51}+\left[Raf\right]}-\frac{k_{r51}\left[Akt*\right]\left[Raf*\right]}{K_{mr51}+\left[Raf*\right]}-\frac{V_{\max r51}\left[Raf*\right]}{K_{mrb51}+\left[Raf*\right]}$
R52	MEK to MEK*	$\frac{k_{f52}\left[Raf*\right]\left[MEK\right]}{K_{mf52}*(1+InH_1/KmI_1)+\left[MEK\right]*(1+a*InH_1/KmI_1)}-\frac{V_{\max r52}\left[MEK*\right]}{K_{mr52}+\left[MEK*\right]}$
R54	SOS to phosphorylated, inactive SOS	$\frac{k_{f54}\left[ERK*\right]\left[O\right]}{K_{mf54}+\left[O\right]}-\frac{V_{\max r54}\left[OP\right]}{K_{mr54}+\left[OP\right]}$
R55	Gab1 to phosphorylated, inactive Gab1	$\frac{k_{f55}\left[ERK*\right]\left[A\right]}{K_{mf55}+\left[A\right]}-\frac{V_{\max r55}\left[AP\right]}{K_{mr55}+\left[AP\right]}$
R56	ErbB1/ErbB3 dimerization	$k_{on56}\left[E-E_1\right]\left[H-E_3\right]-k_{off56}\left[E_{13}\right]$
R57	ErbB1/ErbB4 dimerization	$k_{on57}\left[E-E_1\right]\left[H-E_4\right]-k_{off57}\left[E_{14}\right]$
R58	1-3 phosphorylation	$\frac{k_{f58}\left[E_{13}\right]}{1+InH_3/KmI_3}-\frac{V_{\max 58}\left[E_{13}P\right]}{K_{m58}+\left[E_{13}P\right]}-k_{temp120}\left[\sum T\right]\left[E_{13}P\right]$
R59	1-4 phosphorylation	$\frac{k_{f59}\left[E_{14}\right]}{1+InH_3/KmI_3}-\frac{V_{\max 59}\left[E_{14}P\right]}{K_{m59}+\left[E_{14}P\right]}-k_{temp121}\left[\sum T\right]\left[E_{14}P\right]$
R60	Grb2 binding to E13	$4*k_{on60}\left[G\right]\left[E_{13}P\right]-k_{off60}\left[E_{13}G\right]f_{\sum G}$
R61	Shc binding to E13	$5*k_{on61}\left[S\right]\left[E_{13}P\right]-k_{off61}\left[E_{13}S\right]f_{\sum S}$
R62	PI-3K binding to E13	$3*k_{on62}\left[I\right]\left[E_{13}P\right]-k_{off62}\left[E_{13}I\right]$
R63	RasGAP binding to E13	$2*k_{on63}\left[R\right]\left[E_{13}P\right]-k_{off63}\left[E_{13}R\right]f_{\sum R}$

R64	Grb2 binding to E14	$4 * k_{on64}[G][E_{14}P]-k_{off\,64}[E_{14}G]\,f_{\sum G}$
R65	Shc binding to E14	$6 * k_{on65}[S][E_{14}P]-k_{off\,65}[E_{14}S]\,f_{\sum S}$
R66	PI-3K binding to E14	$1 * k_{on66}[I][E_{14}P]-k_{off\,66}[E_{14}I]$
R67	RasGAP binding to E14	$2 * k_{on67}[R][E_{14}P]-k_{off\,67}[E_{14}R]\,f_{\sum R}$
R68	SOS binding to membrane localized Grb2-Gab1 complex	$k_{on68}\left[\sum G-A\right][O]-k_{off\,68}\left[A-\sum G-O\right]$
R73	PTP-1B binding to E11	$4 * k_{on73}[T][E_{11}P]-k_{off\,73}[E_{11}T]$
R74	PTP-1B binding to E12	$3 * k_{on74}[T][E_{12}P]-k_{off\,74}[E_{12}T]$
R75	PTP-1B binding to E23	$2 * k_{on75}[T][E_{23}P]-k_{off\,75}[E_{23}T]$
R76	PTP-1B binding to E34	$2 * k_{on76}[T][E_{34}P]-k_{off\,76}[E_{34}T]$
R77	PTP-1B binding to E24	$2 * k_{on77}[T][E_{24}P]-k_{off\,77}[E_{24}T]$
R78	PTP-1B binding to E44	$2 * k_{on78}[T][E_{44}P]-k_{off\,78}[E_{44}T]$
R79	PTP-1B binding to E13	$3 * k_{on79}[T][E_{13}P]-k_{off\,79}[E_{13}T]$
R80	PTP-1B binding to E14	$3 * k_{on80}[T][E_{14}P]-k_{off\,80}[E_{14}T]$
R81	ErbB1 phosphorylated, inactive ErbB1	$\frac{k_{kf\,81}[ERK^*][E_1]}{K_{mf\,81}+[E_1]}-\frac{V_{\max r81}[E_1-PT]}{K_{mr81}+[E_1-PT]}$
R82	ErbB2 to phosphorylated, inactive ErbB2	$\frac{k_{kf\,82}[ERK^*][E_2]}{K_{mf\,82}+[E_2]}-\frac{V_{\max r82}[E_2-PT]}{K_{mr82}+[E_2-PT]}$
R83	ErbB4 to phosphorylated, inactive ErbB4	$\frac{k_{kf\,83}[ERK^*][E_4]}{K_{mf\,83}+[E_4]}-\frac{V_{\max r83}[E_4-PT]}{K_{mr83}+[E_4-PT]}$

R84	E-ErbB1 to phosphorylated, inactive E-ErbB1	$\frac{k_{kf84}[ERK*][E-E_1]}{K_{mf84}+[E-E_1]}-\frac{V_{\max r84}[E-E_1-PT]}{K_{mr84}+[E-E_1-PT]}$
R85	H-ErbB4 to phosphorylated, inactive H-ErbB4	$\frac{k_{kf85}[ERK*][H-E_4]}{K_{mf85}+[H-E_4]}-\frac{V_{\max r85}[H-E_4-PT]}{K_{mr85}+[H-E_4-PT]}$
R86	EGF binding to ErbB1-PT	$k_{on86}[E][E_1-PT]/VeVc-k_{off86}[E-E_1-PT]/VeVc$
R87	HRG binding to ErbB4-PT	$k_{on87}[H][E_4-PT]/VeVc-k_{off87}[H-E_4-PT]/VeVc$
R96	ERK binding to MEK*	$k_{on96}[ERK][MEK*]-k_{off96}[ERK-MEK*]$
R97	ERK phosphorylation	$k_{f97}[ERK-MEK*]$
R98	pERK binding to MEK*	$k_{on98}[pERK][MEK*]-k_{off98}[pERK-MEK*]$
R99	pERK phosphorylation	$k_{f99}[pERK-MEK*]$
R100	ERK* binding to ERK Phosphatase	$k_{on100}[ERK*][ERKPase]-k_{off100}[ERK*-ERKPase]$
R101	Dephosphorylation of ERK*	$k_{f101}[ERK*-ERKPase]$
R102	pERK binding to ERK Phosphatase	$k_{on102}[ERKPase][pERK]-k_{off102}[pERK-ERKPase]$
R103	Dephosphorylation of pERK	$k_{f103}[pERK-ERKPase]$
R104	f_{int}	$k_{temp59}*(-f_{\text{int}}+k_{temp60})$
R105	Grb2 binding to Gab1 bound to PIP3	$k_{on57\_2}[P_3-A][G]-k_{off57\_2}[\sum A-G]$
R106	SOS binding to PIP3-Gab1-Grb2 complex	$k_{on58}[O][\sum A-G]-k_{off58}[\sum A-G-O]$
R107	Gab1 binding to membrane localized Grb2-SOS complex	$k_{on59}[A][\sum G-O]-k_{off59}[A-\sum G-O]f_{\sum A}$
R108	PTP-1B binding to membrane localized	$2*k_{on81}[T][\sum AP]-k_{off81}[\sum AP-T]$

	Gab1-P	
R109	phosphorylated E11 degradation	$k_{\deg 10}\left[E_{11}P\right]$
R110	Grb2 binding to E11 degradation	$k_{\deg 10}\left[E_{11}G\right]$
R111	Shc binding to E11 degradation	$k_{\deg 10}\left[E_{11}S\right]$
R112	RasGAP binding to E11 degradation	$k_{\deg 10}\left[E_{11}R\right]$
R113	PTP-1B binding to E11 degradation	$k_{\deg 10}\left[E_{11}T\right]$

VeVc is taken to be 33.3 [Kholodenko et al., 1999]. k_{r41} is 3 and k_{r51} is 0. Units: Concentration (nM), $V_{\max}$ (nM/s), k_{on} (1/s/nM), k_{off} (1/s), K_m (nM), k_f (1/s), k_r (1/s), $k_{\deg}$ (1/s). InH (nM) is inhibitor concentration and KmI (nM) is inhibition constant. if $a > 1$, the inhibition is mixed non-competitive.
