# Supplementary material for: Robustness analysis of the detailed kinetic model of an ErbB signaling network by using dynamic sensitivity
Source: PLoS One. 2017 May 24;12(5):e0178250. doi: 10.1371/journal.pone.0178250 (PMC5443533; doi:10.1371/journal.pone.0178250)
Supplement: S3 Table — (PDF) [file pone.0178250.s003.pdf]

Table S3 Fraction multipliers for seeding molecular species dissociation

Name	***Definition***	***Applicable Reactions***
$f_{\sum G}$	$\sum G/(\sum G+\sum G-O+\sum G-A+A-\sum G-O)$	R16, R19, R22, R26, R30, R34, R42, R60, R64
$f_{\sum S}$	$\sum S/(\sum S+\sum SP+\sum SP-G)$	R17, R20, R23, R27, R31, R35, R43, R61, R65
$f_{\sum R}$	$\sum R/(\sum R+\sum RP)$	R18, R21, R25, R29, R33, R37, R45, R63, R67
$f_{\sum A}$	$\sum A/(\sum A+\sum AP-S+\sum AP-I+\sum AP-R+\sum AP-T)$	R41, R46, R107
