# Supplementary material for: Robustness analysis of the detailed kinetic model of an ErbB signaling network by using dynamic sensitivity
Source: PLoS One. 2017 May 24;12(5):e0178250. doi: 10.1371/journal.pone.0178250 (PMC5443533; doi:10.1371/journal.pone.0178250)
Supplement: S4 Table — (PDF) [file pone.0178250.s004.pdf]

Table S4 Molecular species

Abbreviation	***Description***
E	Epidermal Growth Factor Ligand
H	Heregulin Growth Factor Ligand
E_1	ErbB1 Free Receptor
E_2	ErbB2 Free Receptor
E_3	ErbB3 Free Receptor
E_4	ErbB4 Free Receptor
$E - E_1$	ErbB1 bound to EGF
$H - E_3$	ErbB3 bound to HRG
$H - E_4$	ErbB4 bound to HRG
E_{11}	EGF-ErbB1 dimers
E_{12}	EGF-ErbB1 ErbB2 dimers
E_{23}	HRG-ErbB3 ErbB2 dimers
E_{34}	HRG-ErbB3 HRG-ErbB4 dimers
E_{24}	ErbB2 HRG-ErbB4 dimers
E_{44}	HRG-ErbB4 dimers
$E_{11}P$	Phosphorylated E11
$E_{12}P$	Phosphorylated E12

$E_{23}P$	Phosphorylated E23
$E_{34}P$	Phosphorylated E34
$E_{24}P$	Phosphorylated E2
$E_{44}P$	Phosphorylated E44
G	Free Grb2
S	Free Shc
I	Free PI-3K
R	Free RasGAP
O	Free SOS
A	Free Gab1
$E_{11}G$	1-1 dimer bound to Grb2
$E_{11}S$	1-1 dimer bound to Shc
$E_{11}R$	1-1 dimer bound to RasGAP
$E_{12}G$	1-2 dimer bound to Grb2
$E_{12}S$	1-2 dimer bound to Shc
$E_{12}R$	1-2 dimer bound to RasGAP
$E_{23}G$	2-3 dimer bound to Grb2
$E_{23}S$	2-3 dimer bound to Shc
$E_{23}I$	2-3 dimer bound to PI-3K

$E_{23}R$	2-3 dimer bound to RasGAP
$E_{34}G$	3-4 dimer bound to Grb2
$E_{34}S$	3-4 dimer bound to Shc
$E_{34}I$	3-4 dimer bound to PI-3K
$E_{34}R$	3-4 dimer bound to RasGAP
$E_{24}G$	2-4 dimer bound to Grb2
$E_{24}S$	2-4 dimer bound to Shc
$E_{24}I$	2-4 dimer bound to PI-3K
$E_{24}R$	2-4 dimer bound to RasGAP
$E_{44}G$	4-4 dimer bound to Grb2
$E_{44}S$	4-4 dimer bound to Shc
$E_{44}I$	4-4 dimer bound to PI-3K
$E_{44}R$	4-4 dimer bound to RasGAP
$\sum G$	Membrane localized Grb2
$\sum S$	Membrane localized Shc
$\sum I$	Membrane localized PI-3K
$\sum R$	Membrane localized RasGAP
$\sum A$	Membrane localized Gab1
$\sum SP$	Phosphorylated membrane localized Shc

$\sum AP$	Phosphorylated membrane localized Gab1
$\sum G-O$	SOS bound to membrane bound Grb2
$\sum G-A$	Gab1 bound to membrane bound Grb2
$\sum SP-G$	Grb2 bound to membrane localized, phosphorylated Shc
$\sum AP-S$	Shc bound to membrane localized, phosphorylated Gab1
$\sum AP-I$	PI-3K bound to membrane localized, phosphorylated Gab1
$\sum AP-R$	RasGAP bound to membrane localized, phosphorylated Gab1
P_3-A	Gab1 bound to PIP3
P_2	Free PIP2
P_3	Free PIP3
Akt	Free, inactive Akt
Akt^*	Free, active Akt
RsD	Ras-GDP
RsT	Ras-GTP
$\sum RP$	Membrane localized, phosphorylated RasGAP
Raf	Free, inactive Raf
Raf^*	Free, active Raf
MEK	Free, inactive MEK
MEK^*	Free, active MEK

ERK	Free, inactive ERK
ERK^*	Free, active ERK
OP	Inactive, phosphorylated SOS
AP	Inactive, phosphorylated Gab1
$A-\sum G-O$	Membrane bound Grb2 bound to Gab1 and SOS
$\sum A-G$	Gab1 bound to PIP3 and Grb2
$\sum A-G-O$	Gab1 bound to PIP3 and Grb2-SOS
$\sum O$	Membrane localized SOS
E_{13}	ErbB1-ErbB3 dimers
E_{14}	ErbB1-ErbB4 dimers
$E_{13}P$	Phosphorylated ErbB1-ErbB3 dimers
$E_{14}P$	Phosphorylated ErbB1-ErbB4 dimers
$E_{13}G$	Grb2 bound to ErbB1-ErbB3 dimers
$E_{13}S$	Shc bound to ErbB1-ErbB3 dimers
$E_{13}I$	PI-3K bound to ErbB1-ErbB3 dimers
$E_{13}R$	RasGAP bound to ErbB1-ErbB3 dimers
$E_{14}G$	Grb2 bound to ErbB1-ErbB3 dimers
$E_{14}S$	Shc bound to ErbB1-ErbB3 dimers
$E_{14}I$	PI-3K bound to ErbB1-ErbB3 dimers

$E_{14}R$	RasGAP bound to ErbB1-ErbB3 dimers
f_{int}	Empirical PI-3K fractional multiplier
T	Free PTP-1B
$E_{11}T$	PTP-1B bound to ErbB1 dimers
$E_{12}T$	PTP-1B bound to ErbB1-ErbB2 dimers
$E_{23}T$	PTP-1B bound to ErbB2-ErbB3 dimers
$E_{34}T$	PTP-1B bound to ErbB3-ErbB4 dimers
$E_{24}T$	PTP-1B bound to ErbB2-ErbB4 dimers
$E_{44}T$	PTP-1B bound to ErbB4 dimers
$E_{13}T$	PTP-1B bound to ErbB1-ErbB3 dimers
$E_{14}T$	PTP-1B bound to ErbB1-ErbB4 dimers
$\sum AP-T$	PTP-1B bound to membrane-localized Gab1
$\sum T$	Membrane localized PTP-1B
E_1-PT	Threonine Phosphorylated ErbB1
E_2-PT	Threonine Phosphorylated ErbB2
E_4-PT	Threonine Phosphorylated ErbB4
$E-E_1-PT$	Threonine Phosphorylated ErbB1 bound to EGF
$H-E_4-PT$	Threonine Phosphorylated ErbB4 bound to HRG
$pERK$	Monophosphorylated ERK

$ERK-MEK*$	Unphosphorylated ERK bound to active MEK
$pERK-MEK*$	Monophosphorylated ERK bound to active MEK
$ERKPase$	ERK phosphatase
$ERK*-ERKPase$	Active ERK bound to ERK phosphatase
$pERK-ERKPase$	Monophosphorylated ERK bound to ERK phosphatase
xconc121	
xconc122	
xconc123	
xconc124	
xconc125	
xconc126	
