# Supplementary material for: Robustness analysis of the detailed kinetic model of an ErbB signaling network by using dynamic sensitivity
Source: PLoS One. 2017 May 24;12(5):e0178250. doi: 10.1371/journal.pone.0178250 (PMC5443533; doi:10.1371/journal.pone.0178250)
Supplement: S5 Table — (PDF) [file pone.0178250.s005.pdf]

Table S5 Kinetic parameter list

Kinetic parameter	*value*	*Unit*
$V_{\max 10}$	223.878	nM/s
$V_{\max 11}$	223.878	nM/s
$V_{\max 12}$	223.878	nM/s
$V_{\max 13}$	223.878	nM/s
$V_{\max 14}$	223.878	nM/s
$V_{\max 15}$	223.878	nM/s
$V_{\max 38}$	223.878	nM/s
$V_{\max 39}$	223.878	nM/s
$V_{\max 50}$	223.878	nM/s
$V_{\max 58}$	223.878	nM/s
$V_{\max 59}$	223.878	nM/s
K_{m10}	486.140	nM
K_{m11}	486.140	nM
K_{m12}	486.140	nM
K_{m13}	486.140	nM
K_{m14}	486.140	nM
K_{m15}	486.140	nM

K_{m38}	486.140	nM
K_{m39}	486.140	nM
K_{m50}	486.140	nM
K_{m58}	486.140	nM
K_{m59}	486.140	nM
$k_{\deg 10}$	0.026	$1/s$
k_{f47}	24.605	$1/s$
$V_{\max r47}$	590.506	nM/s
K_{mf47}	698.602	nM
K_{mr47}	483.862	nM
k_{f48}	16.833	$1/s$
K_{mf48}	715.569	nM
K_{mr48}	324.929	nM
$PTEN$	693.579	nM
k_{f49}	44.350	$1/s$
k_{r49}	552.675	$1/s$
K_{mf49}	343.248	nM
K_{mr49}	753.167	nM
K_{mr49b}	381.221	nM

k_{r49b}	640.821	$1/s$
k_{f51}	3.652	$1/s$
$V_{\max r51}$	16.737	nM/s
K_{mf51}	599.708	nM
K_{mr51}	346.478	nM
K_{mrb51}	988.450	nM
k_{f52}	0.774	$1/s$
$V_{\max r52}$	199.277	nM/s
K_{mf52}	545.441	nM
K_{mr52}	675.299	nM
k_{f54}	0.054	$1/s$
$V_{\max r54}$	588.267	nM/s
K_{mf54}	457.965	nM
K_{mr54}	336.183	nM
k_{f55}	0.226	$1/s$
$V_{\max r55}$	646.900	nM/s
K_{mf55}	460.945	nM
K_{mr55}	643.925	nM
k_{f38}	279.993	$1/s$

k_{f39}	385.743	$1/s$
k_{f50}	389.106	$1/s$
k_{temp59}	0.085	-
k_{temp60}	0.183	-
k_{off46}	0.519	$1/s$
k_{off1}	0.018	$1/s$
k_{off86}	0.018	$1/s$
k_{off2}	0.001	$1/s$
k_{off3}	0.097	$1/s$
k_{off87}	0.097	$1/s$
k_{off4}	0.172	$1/s$
k_{off5}	4.398	$1/s$
k_{off6}	2.662	$1/s$
k_{off7}	8.056	$1/s$
k_{off8}	9.103	$1/s$
k_{off9}	5.543	$1/s$
k_{off56}	5.229	$1/s$
k_{off57}	5.514	$1/s$
k_{off16}	0.574	$1/s$

$k_{off\,17}$	4.687	$1/s$
$k_{off\,18}$	2.277	$1/s$
$k_{off\,19}$	2.336	$1/s$
$k_{off\,20}$	0.676	$1/s$
$k_{off\,21}$	4.729	$1/s$
$k_{off\,22}$	3.696	$1/s$
$k_{off\,23}$	2.362	$1/s$
$k_{off\,24}$	4.423	$1/s$
$k_{off\,25}$	2.225	$1/s$
$k_{off\,26}$	0.010	$1/s$
$k_{off\,27}$	1.892	$1/s$
$k_{off\,28}$	4.643	$1/s$
$k_{off\,29}$	2.015	$1/s$
$k_{off\,30}$	4.994	$1/s$
$k_{off\,31}$	1.220	$1/s$
$k_{off\,32}$	3.875	$1/s$
$k_{off\,33}$	1.282	$1/s$
$k_{off\,34}$	3.204	$1/s$
$k_{off\,35}$	1.870	$1/s$

$k_{off\,36}$	1.257	$1/s$
$k_{off\,37}$	0.406	$1/s$
$k_{off\,60}$	0.118	$1/s$
$k_{off\,61}$	2.652	$1/s$
$k_{off\,62}$	1.614	$1/s$
$k_{off\,63}$	2.887	$1/s$
$k_{off\,64}$	3.970	$1/s$
$k_{off\,65}$	2.607	$1/s$
$k_{off\,66}$	2.299	$1/s$
$k_{off\,67}$	0.907	$1/s$
$k_{off\,40}$	3.105	$1/s$
$k_{off\,41}$	7.049	$1/s$
$k_{off\,42}$	3.520	$1/s$
$k_{off\,43}$	0.544	$1/s$
$k_{off\,44}$	0.427	$1/s$
$k_{off\,45}$	3.997	$1/s$
$k_{off\,57\_2}$	0.453	$1/s$
$k_{off\,56}$	6.306	$1/s$
$k_{off\,59}$	9.172	$1/s$

$k_{off\,68}$	4.998	$1/s$
$k_{temp114}$	29.853	$1/s$
$k_{temp115}$	78.204	$1/s$
$k_{temp116}$	11.421	$1/s$
$k_{temp117}$	55.210	$1/s$
$k_{temp118}$	57.751	$1/s$
$k_{temp119}$	60.263	$1/s$
$k_{temp120}$	7.477	$1/s$
$k_{temp121}$	48.634	$1/s$
$k_{off\,73}$	3.005	$1/s$
$k_{off\,74}$	1.250	$1/s$
$k_{off\,75}$	1.432	$1/s$
$k_{off\,76}$	2.154	$1/s$
$k_{off\,77}$	1.224	$1/s$
$k_{off\,78}$	0.201	$1/s$
$k_{off\,79}$	1.185	$1/s$
$k_{off\,80}$	2.937	$1/s$
$k_{temp130}$	83.446	$1/s$
$k_{temp131}$	79.613	$1/s$

k_{off81}	3.925	$1/s$
$k_{temp133}$	96.572	$1/s$
k_{kf81}	1.361	$1/s$
$V_{\max r81}$	242.603	nM/s
K_{mf81}	485.263	nM
K_{mr81}	323.401	nM
k_{kf82}	6.999	$1/s$
$V_{\max r82}$	398.193	nM/s
K_{mf82}	781.437	nM
K_{mr82}	595.839	nM
k_{kf83}	1.763	$1/s$
$V_{\max r83}$	534.053	nM/s
K_{mf83}	609.477	nM
K_{mr83}	653.518	nM
k_{kf84}	4.689	$1/s$
$V_{\max r84}$	634.163	nM/s
K_{mf84}	622.385	nM
K_{mr84}	258.464	nM
k_{kf85}	6.759	$1/s$

$V_{\max r85}$	369.226	nM/s
K_{mf85}	179.649	nM
K_{mr85}	290.767	nM
$k_{temp154}$	9.978	$1/s$
k_{on1}	0.000	$1/s/nM$
k_{on86}	0.004	$1/s/nM$
k_{on2}	0.006	$1/s/nM$
k_{on3}	0.033	$1/s/nM$
k_{on87}	0.001	$1/s/nM$
k_{on4}	0.500	$1/s/nM$
k_{on5}	2.543	$1/s/nM$
k_{on6}	0.228	$1/s/nM$
k_{on7}	1.061	$1/s/nM$
k_{on8}	1.026	$1/s/nM$
k_{on9}	2.287	$1/s/nM$
k_{on56}	0.805	$1/s/nM$
k_{on57}	1.782	$1/s/nM$
k_{f10}	0.650	$1/s$
k_{f11}	0.372	$1/s$

k_{f12}	1.801	$1/s$
k_{f13}	0.887	$1/s$
k_{f14}	6.173	$1/s$
k_{f15}	1.356	$1/s$
k_{f58}	0.930	$1/s$
k_{f59}	1.208	$1/s$
k_{on16}	0.010	$1/s/nM$
k_{on17}	0.005	$1/s/nM$
k_{on18}	0.012	$1/s/nM$
k_{on73}	0.012	$1/s/nM$
k_{on19}	0.090	$1/s/nM$
k_{on20}	0.048	$1/s/nM$
k_{on21}	0.011	$1/s/nM$
k_{on74}	0.013	$1/s/nM$
k_{on22}	0.001	$1/s/nM$
k_{on23}	0.014	$1/s/nM$
k_{on24}	0.005	$1/s/nM$
k_{on25}	0.100	$1/s/nM$
k_{on75}	0.014	$1/s/nM$

k_{on26}	0.036	$1/s/nM$
k_{on27}	0.020	$1/s/nM$
k_{on28}	0.007	$1/s/nM$
k_{on29}	0.035	$1/s/nM$
k_{on76}	0.005	$1/s/nM$
k_{on30}	0.002	$1/s/nM$
k_{on31}	0.003	$1/s/nM$
k_{on32}	0.001	$1/s/nM$
k_{on33}	0.033	$1/s/nM$
k_{on77}	0.010	$1/s/nM$
k_{on34}	0.000	$1/s/nM$
k_{on35}	0.060	$1/s/nM$
k_{on36}	0.004	$1/s/nM$
k_{on37}	0.079	$1/s/nM$
k_{on78}	0.008	$1/s/nM$
k_{on60}	0.012	$1/s/nM$
k_{on61}	0.000	$1/s/nM$
k_{on62}	0.000	$1/s/nM$
k_{on63}	0.004	$1/s/nM$

k_{on79}	0.008	$1/s/nM$
k_{on64}	0.008	$1/s/nM$
k_{on65}	0.012	$1/s/nM$
k_{on66}	0.016	$1/s/nM$
k_{on67}	0.035	$1/s/nM$
k_{on80}	0.000	$1/s/nM$
k_{on40}	0.019	$1/s/nM$
k_{on41}	0.005	$1/s/nM$
k_{on42}	0.002	$1/s/nM$
k_{on43}	0.013	$1/s/nM$
k_{on44}	0.012	$1/s/nM$
k_{on45}	0.003	$1/s/nM$
k_{on81}	0.011	$1/s/nM$
k_{on46}	0.015	$1/s/nM$
k_{on57_2}	0.004	$1/s/nM$
k_{on58}	0.022	$1/s/nM$
k_{on59}	0.008	$1/s/nM$
k_{on68}	0.000	$1/s/nM$
k_{on96}	0.200	$1/s/nM$

$k_{off\,96}$	99.964	$1/s$
$k_{f\,97}$	20.004	$1/s$
k_{on98}	0.197	$1/s/nM$
$k_{off\,98}$	99.998	$1/s$
$k_{f\,99}$	0.200	$1/s$
k_{on100}	0.200	$1/s/nM$
$k_{off\,100}$	100.004	$1/s$
$k_{f\,101}$	0.997	$1/s$
k_{on102}	0.199	$1/s/nM$
$k_{off\,102}$	100.002	$1/s$
$k_{f\,103}$	19.985	$1/s$
