# Supplementary material for: Robustness analysis of the detailed kinetic model of an ErbB signaling network by using dynamic sensitivity
Source: PLoS One. 2017 May 24;12(5):e0178250. doi: 10.1371/journal.pone.0178250 (PMC5443533; doi:10.1371/journal.pone.0178250)
Supplement: S6 Table — (PDF) [file pone.0178250.s006.pdf]

Table S6 Initial concentration of molecular species

Species	*Value*
ErbB1	274
ErbB2	158
ErbB3	294
ErbB4	399
Grb2	82.4
Shc	11.5
PI-3K	46.4
RasGAP	93.9
PTP-1B	500
SOS	82.3
Gab1	43.1
PIP2	197
Akt	444
Ras	95.7
Raf	743
MEK	772
ERK	750
ERK-Ppase	35

All variables not shown in this table are set to zero or $1x10^{-14}$. These initial concentrations are derived from protein abundances. All units are nM. The ligand (HRG and EGF) initial concentrations are systematically changed.
